# Supplementary material for: Titanium Dioxide, but Not Zinc Oxide, Nanoparticles Cause Severe Transcriptomic Alterations in T98G Human Glioblastoma Cells
Source: Int J Mol Sci. 2021 Feb 19;22(4):2084. doi: 10.3390/ijms22042084 (PMC7923231; doi:10.3390/ijms22042084)
Supplement: Supplementary file 1 [file ijms-22-02084-s001.zip › ijms-1075533 proofed sup/Supplementarty material/ijms-1075533-supplementary corrected.pdf]

Supplementary Materials:

Contents:

Figure S1: The Z-potential analysis of NPs in type 1 water……………………………….. 1
Figure S2: T98G human glioblastoma cells visualized by SEM…………………………. 2
Figure S3 Examples of the electropherograms used to estimate the RIN in the samples used for RNAseq……………………………………………………………………….. 3
Figure S4: Preliminary physical characterization of the TiO_2-NPs and ZnO-NPs………… 3
Table S1 Gross RNAseq experiment data………………………………………………… 4
Table S2: Differentially expressed genes………………………………………………….. 5
Table S3: List of the genes expressed by T98G cells……………………………………... 5
Table S4: Proteomic raw data…………………………………………………………….. 5
Table S5: Proteins whose expressions were statistically altered after exposure to TiO_2-NPs……………………………………………………………………………………….. 6
Table S6: Effect of NPs on the expressions of the different human housekeeping genes… 7

A

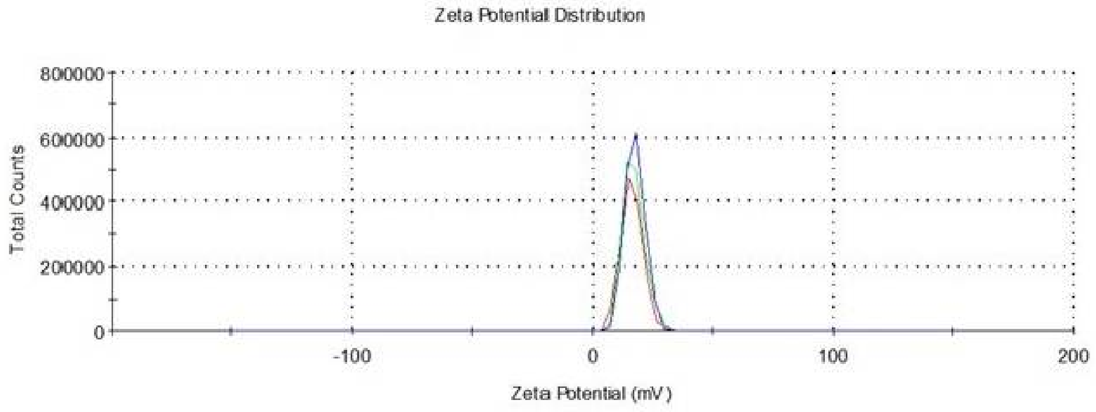

B

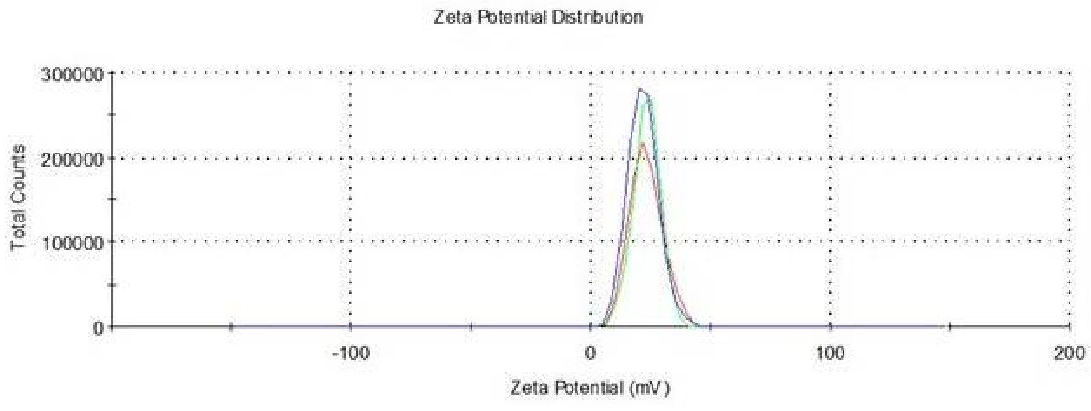

Figure S1. The Z-potential analysis of NPs in type 1 water**.** The three independent runs are displayed. The mean value is +17.0 ± 0.6 mV for ZnO-NPs (panel A) and +22.8 ± 0.8 mV for TiO_2-NPs (panel B).

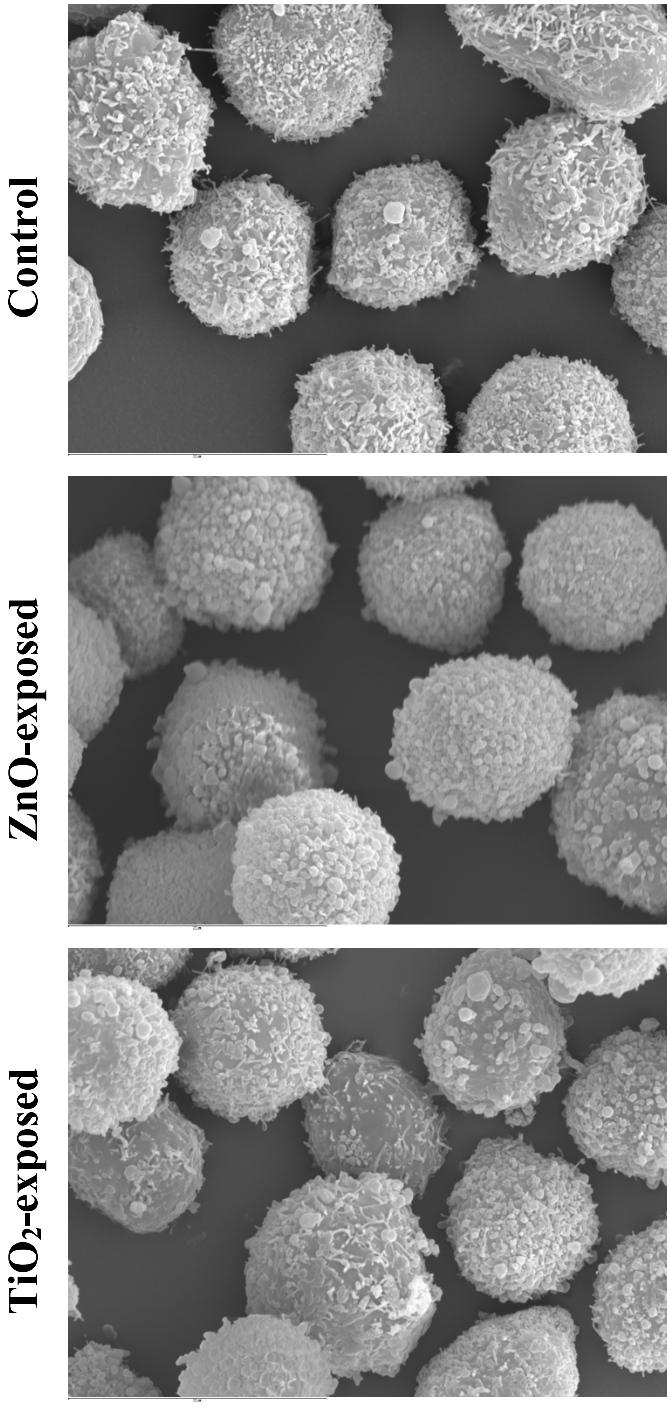

Figure S2. T98G human glioblastoma cells visualized by SEM. Cells were exposed to 20 µg/ml of TiO_2-NPs or 5 µg ZnO-NPs for 72 h, as described in Section 4.4, and then prepared for SEM microscopy (2500x), as described in Section 4.7.

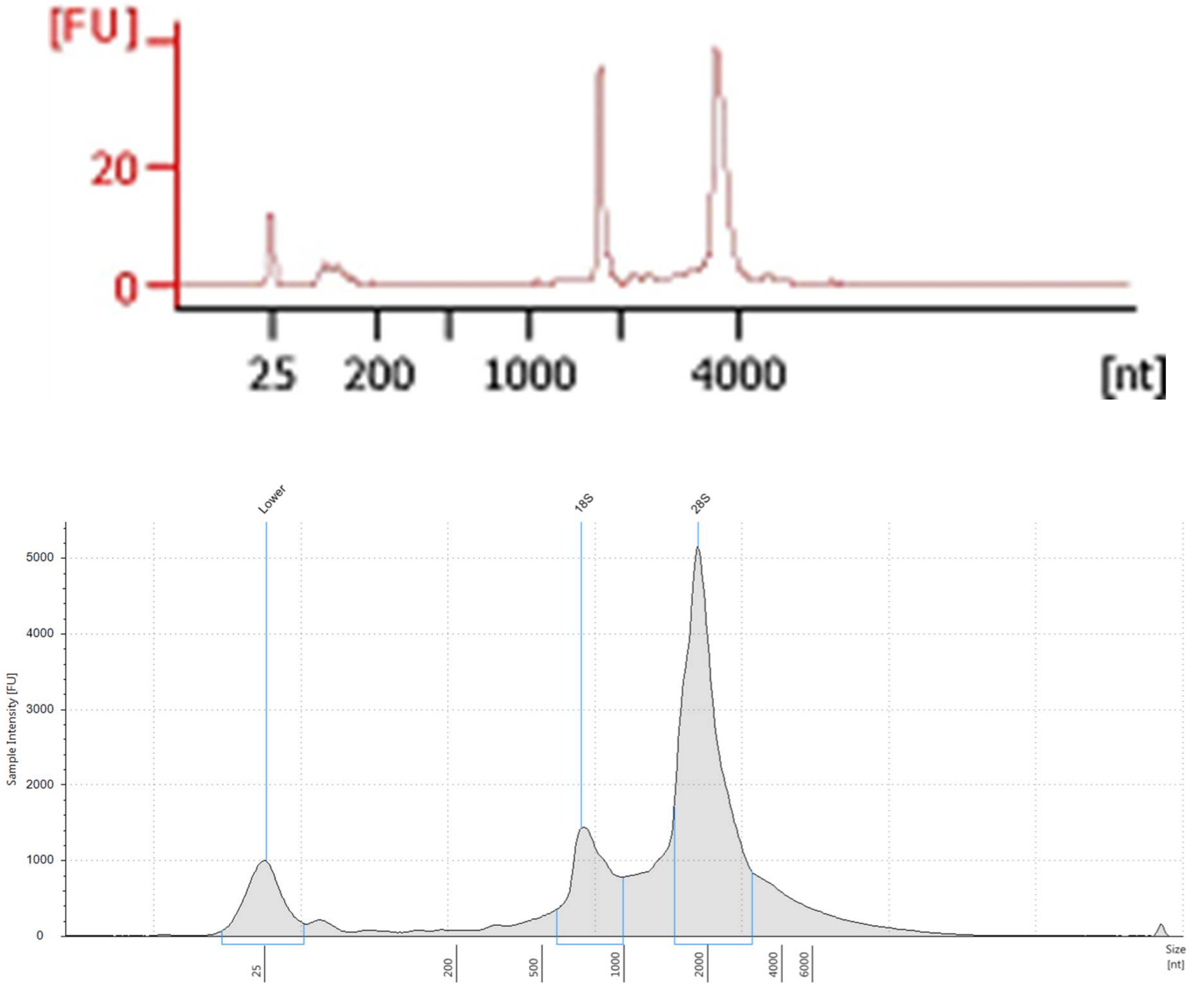

Figure S3. Examples of the electropherograms used to estimate the RIN in the samples used for RNAseq. The top picture corresponds to the RNA of replicate 1 of the cells exposed to the TiO_2 nanoparticles obtained immediately after RNA isolation and corresponds to a RIN of 9.8. The bottom picture corresponds to the same sample obtained by Macrogen Inc. and corresponds to a RIN of 7.8.

TiO_2-NPs **ZO-NPs**

A **B**

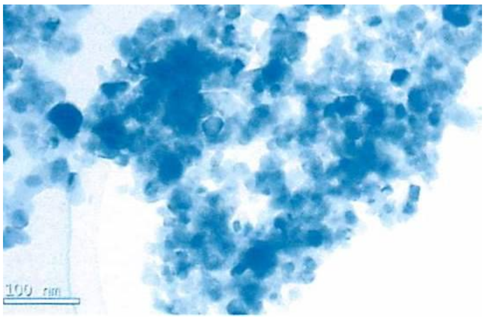

C

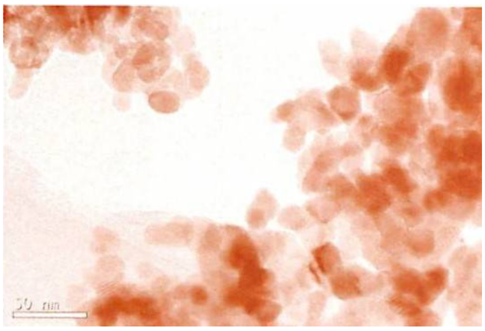

D

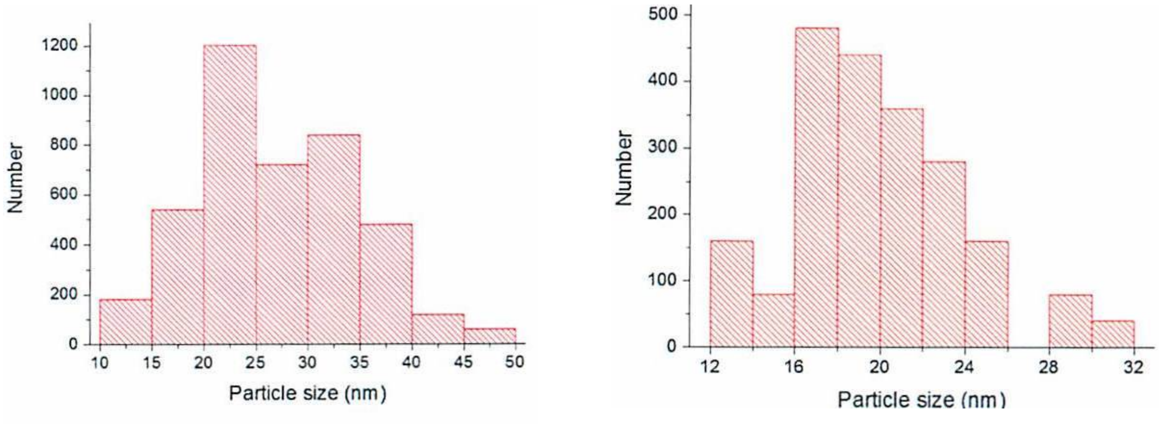

Figure S4. Preliminary physical characterizations of the TiO2-NPs and ZnO-NPs. The TEM images (**A**,**B**) and distribution sizes (**C**,**D**) were provided by the supplier.

Table S1. Gross RNAseq experiment data. These experiments were run in parallel to the silver NPs and shared controls with the data published by Fuster and coworkers (2020).

Sample	Number of Reads	Number of Nucleotides	GC (%)	Q20 (%)	Q30 (%)
Control 1	31,016,624	3,132,679,024	50.1	98.5	95.6
Control 2	30,419,346	3,072,353,946	50.3	98.6	95.7
Control 3	32,022,288	3,234,251,088	49.8	98.5	95.6
ZnO-NPs 1	34,746,146	3,509,360,746	50.0	98.5	95.4
ZnO-NPs 2	35,408,740	3,576,282,740	49.5	98.3	95.2
ZnO-NPs 3	41,848,010	4,226,649,010	50.8	98.0	94.8
TiO_2-NPs 1	40,538,138	4,094,351,938	49.7	98.6	95.7
TiO_2-NPs 2	37,085,974	3,745,683,374	49.7	98.6	95.4
TiO_2-NPs 3	34,221,882	3,456,410,082	50.5	98.4	95.3

Table S2: Differentially expressed genes. The statistical analysis was assessed by considering a 5% FDR.

Table S2
TiO2DEG.xls

Table S3: List of the genes expressed by T98G cells. The list of genes was obtained from the control unexposed cells used in the RNAseq experiment. This list of genes was used as a control for the annotations of the differentially expressed genes after exposure to TiO_2-NPs using the PANTHER tool of the GO Consortium tools. These experiments were run in parallel to the silver NPs and shared controls with the data published by Fuster and coworkers [15].

Table S3 Reference
list.xls

Table S4: Proteomic raw data.

Table S4 protemoic
raw.xlsx

Table S5. Proteins whose expressions statistically altered after the exposure to TiO_2-NPs. The treated/control ratio was calculated using the total intensity signal of each protein for both the treated and untreated cells. The statistical significance was estimated using mean ± SD for the three biological replicates and a Student's *t*-test. * = not detected in the treated cells. ** = not detected in the control cells.

UniProt Database Entry	Name	Treated/Control Ratio	Statistical Significance
	Cytoplasmic proteins		
P04406	Glyceraldehyde-3-phosphate dehydrogenase	0.66	$p < 0.01$
P04075	Fructose-bisphosphate aldolase A	0.70	$p < 0.01$
Q04828	Aldo-keto reductase family 1 member C1	0.42	$p < 0.01$
B4DK69	Aldo-keto reductase family 1 member C2	0.45	$p < 0.001$
A0A0A0MSS8	Aldo-keto reductase family 1 member C3	*	-
A0A384NPQ2	Epididymis secretory sperm binding protein	0.70	$p < 0.01$
O60218	Aldo-keto reductase family 1 member B10	0.42	$p < 0.01$
B2RAR6	Eukaryotic translation elongation factor 1 delta (Guanine nucleotide exchange protein), isoform CRA_b	0.74	$p < 0.05$
A0A0U1RQF0	Fatty acid synthase	0.63	$p < 0.05$
A0A384MTL2	Transgelin	0.58	$p < 0.01$
A0A024R1N1	Myosin, heavy polypeptide 9, non-muscle, isoform CRA_a	2.00	$p < 0.05$
D6RE83	Ubiquitin carboxyl-terminal hydrolase	1.35	$p < 0.05$
A0A087WVQ6	Clathrin heavy chain	2.34	$p < 0.05$
Q13501	Sequestosome-1	1.95	$p < 0.05$
A0A024R592	Glucosidase, alpha neutral AB, isoform CRA_b	1.92	$p < 0.01$
P17987	T-complex protein 1 subunit alpha	0.63	$p < 0.05$
P14174	Macrophage migration inhibitory factor	2.29	$p < 0.05$
Q2XPP3	Type II 3a-hydroxysteroid dehydrogenase variant	**	-
A0A024RC65	HCG1991735, isoform CRA_a	**	-
	Membrane proteins		
P04406	Glyceraldehyde-3-phosphate dehydrogenase	0.33	$p < 0.01$
B4E335	Actin, cytoplasmic 1	0.60	$p < 0.05$
B7Z6P1	Actin, alpha cardiac muscle 1	0.46	$p < 0.05$
B3KTV0	Heat shock cognate 71-kDa protein	0.63	$p < 0.05$
P14625	Endoplasmin	0.47	$p < 0.05$
A8K486	Peptidyl-prolyl cis-trans isomerase	0.63	$p < 0.001$
A0A384NPQ2	Epididymis secretory sperm-binding protein	0.44	$p < 0.05$
Q13162	Peroxiredoxin-4	0.24	$p < 0.001$
A0A087WVQ9	Elongation factor 1-alpha 1	0.47	$p < 0.05$
J3KPS3	Fructose-bisphosphate aldolase	0.46	$p < 0.05$
B4DK69	Aldo-keto reductase family 1 member C2	0.82	$p < 0.05$
P04792	Heat shock protein beta-1	0.39	$p < 0.05$
E9PK25	Cofilin-1	0.60	$p < 0.01$
A0A024R5Z7	Annexin	0.41	$p < 0.05$
B7ZW00	COL6A3 protein	0.65	$p < 0.05$
A0A024RDR0	High-mobility group box 1, isoform CRA_a	0.34	$p < 0.001$
P30050	60S ribosomal protein L12	0.37	$p < 0.05$
A2RUM7	Ribosomal protein L5	0.38	$p < 0.05$
C9J4W5	Eukaryotic translation initiation factor 5A	0.55	$p < 0.01$
A0A024R2Q4	Ribosomal protein L15	0.20	$p < 0.01$
A0A140VJX1	Testicular tissue protein Li 198	0.16	$p < 0.01$
B4DM94	60S ribosomal protein L18a	0.54	$p < 0.05$
P26038	Moesin	0.36	$p < 0.01$
B7Z4R3	T-complex protein 1 subunit beta	0.05	$p < 0.05$
P60866	40S ribosomal protein S20	0.56	$p < 0.05$
B7Z6Z4	Myosin light polypeptide 6	0.43	$p < 0.05$
H9ZYJ2	Thioredoxin	0.15	$p < 0.01$

A0A2R8Y623	40S ribosomal protein S7	0.47	$p < 0.001$
A0A024RBS2	60S acidic ribosomal protein P0	0.19	$p < 0.01$
A0A024R608	Ribosomal protein, large, P1, isoform CRA_a	0.50	$p < 0.05$
A0A140VK93	Adenylate kinase 2, mitochondrial	0.28	$p < 0.05$
A0A0A0MTS2	Glucose-6-phosphate isomerase	0.22	$p < 0.05$
Q04828	Aldo-keto reductase family 1 member C1	*	-
E7EMC7	Sequestosome-1	*	-
Q13501	Sequestosome-1	**	-
A0A0B4J2C3	Translationally controlled tumor protein	*	-
A0A024RDY2	Tumor protein, translationally controlled 1, isoform CRA_a	**	-

Table S6. Effects of NPs on the expressions of the different human housekeeping genes. T98G human glioblastoma cells were exposed to 20 µg/ml of TiO_2-NPs for 72 h, as described in Section 4.4. Then, RNA was isolated as described in Section 4.8 to be reverse-transcribed and quantified by RT-PCR, as described in Section 4.11. The mean and standard deviation (SD) of the number of thermal cycles for three independent wells per experimental condition are displayed. * = statistically different from the control for at least $p<0.05$. The genes finally selected for the PCR experiments are shadowed. These experiments were run in parallel to the silver NPs and shared controls with the data published in [15].

	**Control**		**Ag NPs**	
**Genes**	**Mean**	**SD**	**Mean**	**SD**
18S rRNA	12.19	0.28	15.81*	0.33
GAPDH	15.46	0.44	15.69	0.48
HPRT1	22.45	0.78	22.64	0.10
GUSB	22.36	0.40	22.58	0.29
ACTB	18.87	0.38	19.32	0.58
B2M	18.12	0.11	18.14	0.17
HMBS	22.85	0.35	23.04	0.11
IPO8	25.52	0.35	25.84	0.24
PGK1	18.73	0.38	18.31	0.31
RPLP0	18.29	0.46	17.99	0.01
TBP	26.13	0.28	25.66	0.10
TFRC	20.97	0.23	21.37	0.16
UBC	18.97	0.34	18.32*	0.08
YWHAZ	26.47	0.50	26.17	0.12
PPIA	17.23	0.43	16.73	0.17
POLR2A	28.21	0.57	28.40	0.32
CASC3	23.75	0.31	24.21	0.27
CDKN1A	27.47	0.45	27.19	0.25
CDKN1B	24.83	0.32	25.08	0.15
GADD45A	27.12	0.67	25.02*	0.39
PUM1	24.59	0.34	25.24	0.23
PSMC4	23.40	0.52	23.19	0.21
EIF2B1	23.80	0.42	23.83	0.18
PES1	22.79	0.31	22.58	0.25
